# A Novel Functionalized MoS_2_-Based Coating for Efficient Solar Desalination

**DOI:** 10.3390/ma16083105

**Published:** 2023-04-14

**Authors:** Qinghong Yu, Qingmiao Wang, Tao Feng, Li Wang, Zhixuan Fan

**Affiliations:** 1 College of Resources and Environmental Engineering, Wuhan University of Science and Technology, Wuhan 430081, China; 2 Center of Green Control and Remediation Technologies for Environmental Pollution, Wuhan University of Science and Technology, Wuhan 430081, China

**Keywords:** molybdenum disulfide, functionalization, coatings, solar desalination

## Abstract

Molybdenum disulfide (MoS_2_) has emerged as a promising photothermal material for solar desalination. However, its limitation in integrating with organic substances constrains its application because of the lack of functional groups on its surface. Here, this work presents a functionalization approach to introduce three different functional groups (-COOH -OH -NH_2_) on the surface of MoS_2_ by combining them with S vacancies. Subsequently, the functionalized MoS_2_ was coated on the polyvinyl alcohol-modified polyurethane sponge to fabricate a MoS_2_-based double-layer evaporator through an organic bonding reaction. Photothermal desalination experiments show that the functionalized material has higher photothermal efficiency. The evaporation rate of the hydroxyl functionalized the MoS_2_ evaporator evaporation rate is 1.35 kg m^−2^ h^−1^, and the evaporation efficiency is 83% at one sun. This work provides a new strategy for efficient, green, and large-scale utilization of solar energy by MoS_2_-based evaporators.

## 1. Introduction

Freshwater scarcity is one of the most challenging issues of the 21st century, and with 96.5% of the Earth’s surface water being seawater, desalination is an ideal solution [1]. Common desalination methods include membrane filtration [2], electrochemical methods [3,4], and reverse osmosis methods [5]. However, these methods suffer from high energy consumption, high cost, and high infrastructure requirements [6]. Solar energy is a sustainable approach to manufacturing fresh water since it is a renewable and clean energy source that can heat liquid water to make steam [7].

Photothermal materials significantly impact the rate at which solar energy is utilized [8]. Metal nanoparticles [9,10,11], carbon-based materials [12], semiconductors [13], and polymer-based materials [14] are currently the most widely used photothermal materials. However, these materials have complicated preparation procedures, require high temperatures, and have problems, such as byproduct generation, high generation costs, and low solar energy conversion efficiency [15,16,17].

In recent years, MoS_2_ has been considered an ideal material for photothermal conversion because of its abundant earth reserves, stable chemical properties, and excellent light-absorbing properties [18,19,20,21]. Deoukchen Ghim et al. first investigated MoS_2_ as a potential functional material for solar steam generation [22,23]. Wang et al. constructed a bilayer structure using a thick insulating porous matrix loaded with MoS_2_ to achieve interfacial heating for seawater desalination [24]. However, the MoS_2_ nanosheets made from molybdenite as the raw material via electrochemical liquid exfoliation lack any organic functional groups and are challenging to combine with organic materials, which restricts their potential [25,26]. The introduction of functional groups on the surface of MoS_2_ by function with thiol-containing compounds is a commonly used method [27,28,29], which is based on the formation of covalent bonds between the S atoms and the metal atoms on the surface [30]. Chou et al. first investigated the complex reaction by S-Vacancy chemical coupling of thiol ligands with MoS_2_ nanosheets [31]. Zhou et al. adjusted the amount of introduced organic functional groups by changing the experimental conditions [32]. Wu et al. modulate the electronic system and photothermal properties of MoS_2_ by changing the number of S vacancies on its surface to perform efficient photocatalysis [33]. In this study, three hydrophilic functional groups were introduced on the surface of MoS_2_ by three double-group thiol compounds.

In addition, the current procedure of preparing a photothermal evaporator by loading MoS_2_ onto a substrate is complicated; photothermal evaporation by inking the photothermal material onto the substrate is a simple and efficient process. Zhang et al. made a double-layer structured evaporator by loading Chinese ink onto a wood substrate at an evaporation rate of 1.6 kg m^−2^ h^−1^ at one sun [34]. Jiang et al. prepared bilayer MoS_2_/LaF_3_/PDMS-PTFE membranes by spraying method for efficient solar energy harvesting and simple water treatment with an evaporation rate of 1.76 kg m^−2^ h^−1^ photothermal conversion efficiency up to 91% [35]. Chen et al. achieved a stable ink by encapsulating Chinese ink in a calcium alginate framework at a simple and low cost and then loaded the ink on different substrates for the photothermal desalination photothermal evaporation rate of 1.44 kg m^−2^ h^−1^ and the photothermal conversion rate of 88.05% [36]. Based on the previously functionalized molybdenum disulfide, a binder containing the corresponding functional groups is selected to react to form stable coatings, which is then loaded onto the surface of polyurethane sponge modified with polyvinyl alcohol by dip coating to form a stable self-floating double layer evaporator.

In this paper, we introduce functional groups on the surface of MoS_2_ through functionalization and then react the functionated MoS_2_ with organic substances to form a stable composite structure to achieve coating. The coating of functionalized MoS_2_ is loaded on the surface of a polyurethane sponge by the dipping method to make a photothermal evaporator, thus enabling efficient, low-cost, and large-scale preparation of photothermal evaporators for solar steam generation.

## 2. Results and Discussions

### 2.1. Optimization of Experimental Conditions for the Preparation of MoS_2_

**Figure 1:**
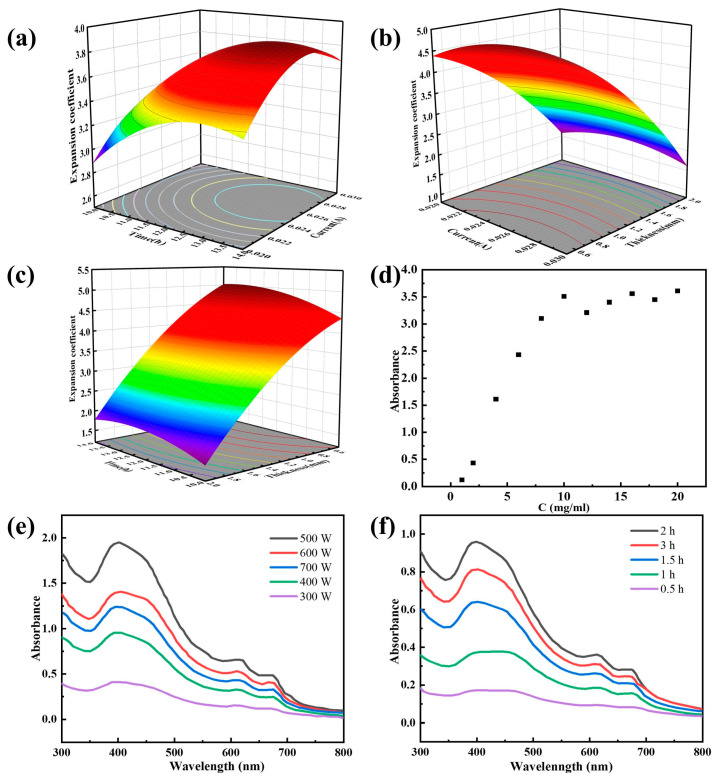
Response surface diagrams of expansivity as a function of (**a**) time and current, (**b**) current and thickness, and (**c**) time and thickness. UV-Vis of MoS_2_ was obtained by exfoliation at different initial concentrations (**d**), ultrasonic power (**e**), and ultrasonic time (**f**).

The influence of electrolysis time and current on the molybdenite expansion coefficient during electrolysis was explored and found to be linear growth in general. (Figure S1). Figure 1a–c shows that the expansion coefficient of molybdenite increases dramatically with increasing current and time, which is consistent with prior experimental results for the conditions (Table S1). However, when the thickness of MoS_2_ increases, the expansion coefficient decreases because the thicker the molybdenite, the stronger the van der Waals forces must resist, and the optimal circumstances are eventually attained. (12.884 h 0.025 A 0.518 mm) (Table S2).

To investigate the influence of initial concentration on exfoliation, different concentrations of MoS_2_ nanosheet dispersions were prepared by taking varying masses of expanded molybdenite flakes and adding the same volume of IPA solvent for ultrasonic. At various initial concentrations of molybdenite, liquid phase exfoliation (500 W, 2 h, 8000 rpm) was conducted, and the supernatant was diluted to a particular multiple after centrifugation. Figure 1d shows the UV-vis spectra of MoS_2_ nanosheet dispersions obtained by evaluating different initial molybdenite concentrations. It can be seen that when the initial molybdenite concentration increases, the attention of the dispersion grows and then decreases, which is because the number of nanosheets formed by exfoliation is limited when the initial concentration is too low. When the initial concentration is too high, it weakens the ultrasonic shear energy and causes the number of exfoliated nanosheets to decrease. Finally, the ideal initial MoS_2_ concentration was 10 mg/mL.

Also investigated were the effects of ultrasonic treatment time and intensity on exfoliation. The ultrasonic power for the probe ultrasonic was set to 300 W, 400 W, 500 W, 600 W, and 700 W for a duration of 1 h. The resulting dispersions were centrifuged, and the supernatant liquid was collected to obtain MoS_2_ nanosheet dispersions prepared at various concentrations for testing the UV-vis spectra. As shown in Figure 1e, as the ultrasonic power increased, the concentration of the dispersed material first grew and then fell. This is related to the ultrasound cavitation effect [37]. When the ultrasonic power is insufficient, the shear force and cavitation of the exfoliated MoS_2_ particles are weak, and it is difficult to overcome the van der Waals force between the MoS_2_ layers, resulting in a low level of exfoliation. A rise in ultrasonic emission is indicative of transient cavitation. Cavitation bubbles increase, but effective bubbles decrease [38], thereby diminishing knockdown strength. Therefore, 500 W is the optimal ultrasound power.

The influence of ultrasonic time on the fabrication of MoS_2_ nanosheets was examined. Under conditions of 500 W power and ultrasonic times of 0.5, 1, 1.5, 2, and 3 h, the concentration of the obtained MoS_2_ nanosheets was examined. The UV spectrum in Figure 1f shows that absorbance increases over time and decreases after three hours. This may be due to the oxidation of MoS_2_ edges brought on by protracted ultrasonic [39], which causes agglomeration and subsequently reduces the concentration. Two hours is optimal for this investigation. The presence of two characteristic absorption peaks at 610 nm and 672 nm, only detected in two-dimensional layered MoS_2_ nanosheets [40], indicates that molybdenite was successfully used to exfoliate MoS_2_ nanosheets.

### 2.2. Characterization

**Figure 2:**
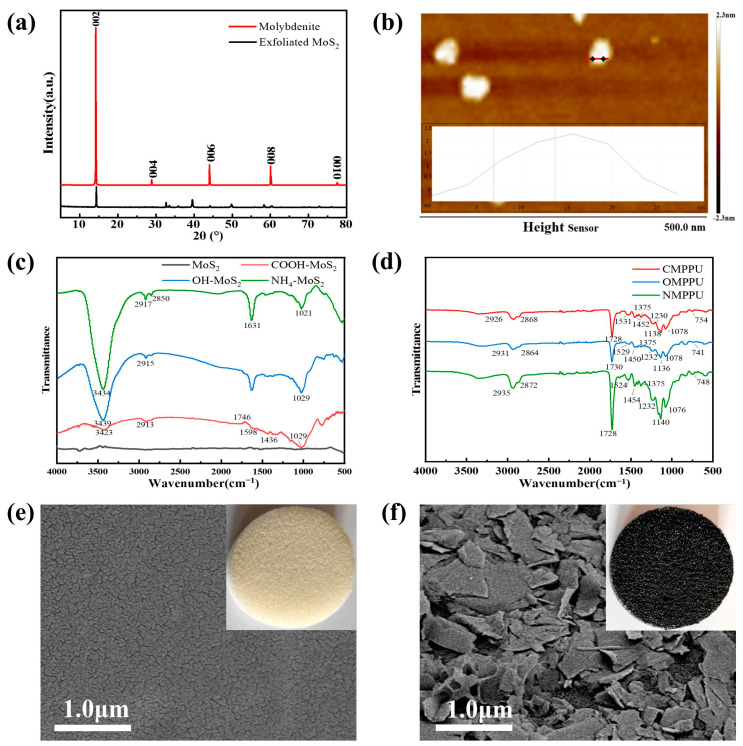
(**a**) XRD spectrum and (**b**) AFM images of the exfoliated MoS_2_. FTIR spectrum of (**c**) functionalization MoS_2_ and (**d**) load functionalization MoS_2_ PPU. (**e**) SEM images of raw PU sponge and (**f**) MoS_2_ supported on PU sponge.

The crystal structure of molybdenite before and after exfoliation was analyzed by XRD. As illustrated in Figure 2a, the diffraction angles of molybdenite at 14.2°, 29.0°, 44.1°, 60.1°, and 77.6° correspond to (002), (004), (006), (008), and (0100) of MoS_2_, respectively. The symmetrical and sharp diffraction peaks of the crystal plane indicate that molybdenite has a high degree of crystallinity. The reduction in the intensity of the MoS_2_ diffraction peaks obtained after exfoliation is due to the reduction in the number of layers compared to the original molybdenite, and the absence of diffraction peaks of other substances indicates that the generated MoS_2_ was relatively pure.

The AFM pattern of MoS_2_ nanosheets derived by centrifuging exfoliated molybdenite is depicted in Figure 2b. The MoS_2_ layer has a thickness of approximately 1.036 nm, which is slightly thicker than that of the monolayer MoS_2_ (0.8 nm) [41], likely owing to the presence of adsorbent on the nanosheets [42]. The AFM results indicate that MoS_2_ nanosheets have been successfully produced by the electrochemical liquid exfoliation of molybdenite.

On the surface of MoS_2_, three functional groups consisting of hydroxyl, amino, and carboxyl groups were introduced through the reaction of sulfhydryl groups with MoS_2_ S vacancies. Before testing FTIR, four distinct samples were placed in Petri dishes and oven-dried at 60 °C for six hours. As shown in Figure 2c, the obtained MoS_2_ nanosheets have no obvious functional group. None of the three functionalized samples exhibited a peak at 2551 cm^−1^ [43], indicating that the -SH of organics effectively reacted with MoS_2,_ and no organics remained. [44]. Compared with the exfoliated MoS_2_, the new characteristic absorption peaks at 1746 cm^−1^, 1598 cm^−1^, and 1436 cm^−1^ correspond to the C=O stretching vibration peak of -COOH, the bending vibration of C=C and -CH_2_ peak. 3423 cm^−1^ and 1029 cm^−1^ correspond to the -OH stretching vibration peak and C-O stretching vibration of -COOH [45]. The presence of hydroxyl groups is indicated by the presence of a broad and strong peak at 3439 cm^−1^. The bending vibration peak of C-H- corresponds to 2915 cm^−1^. The antisymmetric stretching vibration of -CH_2_ is 2913 cm^−1^, while the stretching vibration of C-O is 1028 cm^−1^ [46]. A total of 3434 cm^−1^ is the stretching vibration of -NH_2_, 1631 cm^−1^ is caused by the bending vibration of N-H, and 1021 cm^−1^ is the stretching vibration of C-N. The peaks at 2917 cm^−1^ and 2850 cm^−1^ are bending vibration peaks of C-H [47]. The carboxyl function effect is the lowest in terms of peak intensity, while the amino function effect is the greatest. This is because the surface of MoS_2_ has a negative charge, the carboxyl group repels hydroxyl electronegativity, which is weaker than carboxyl electronegativity, the functional effect is superior, and the amino group combines best with MoS_2_ due to its positive electrical charge [48].

Figure 2d depicts the FTIR images of PPU supplied with three different functional groups of functionalized MoS_2_. The absorption peaks from 2926 to 2935 cm^−1^ are caused by the C-H asymmetric stretching vibration, and the absorption from 2864 to 2872 cm^−1^ is caused by the C-H symmetric stretching vibration. 1728–1730 cm^−1^ is attributed to the C=O elongation oscillation. The absorption peak near 1500 cm^−1^ is caused by the backbone of the aromatic ring framework vibrations. 1450 cm^−1^ and 1375 cm^−1^ are caused by C-H asymmetric bending vibration and C-H symmetric bending vibrations, respectively; the absorption peaks near 1232 cm^−1^ and 1140 cm^−1^ are induced by the antisymmetric stretching vibration of C-O-C and the symmetric stretching vibration of C-O-C, respectively. The bending vibration of the benzene ring within the hydrocarbon plane on the benzene ring is what causes the absorption peak at 1076 cm^−1^, and the absorption peak around 750 cm^−1^ is caused by the out-of-plane bending vibration of hydrocarbons on the benzene ring. It can be found that the absorption peaks previously observed near 3400 cm^−1^ all disappear [49], which is due to the reaction of three functional groups of carboxyl, hydroxyl, and amino groups on the surface of MoS_2_ with the organic matter in the solvent, which, in turn, is immobilized on PPU.

The surface morphology of MoS_2_ nanoparticles before and after loading on a polyurethane sponge is shown in Figure 2e,f, respectively. We found that the primitive sponge surface was rough and wrinkled, and the MoS_2_ was embedded in it after loading on the surface, as shown in Figure 2f. The binder binds MoS_2_ firmly together, and the MoS_2_ accumulated on the surface increases the light area and improves the light utilization rate, and the loading of MoS_2_ solution without binder is obviously weaker than that of the coating (Figure S2). After loading the functionalized MoS_2_ coating, the PPU changes from white to black, which is conducive to light absorption. Figure S3 shows the before and after loading of MoS_2_, demonstrating the evaporator’s remarkable mechanical stability and folding characteristics.

### 2.3. Solar Desalination Experiment

MoS_2_ coatings were applied to PVA-modified polyurethane sponges (PPU) to prepare functionalized MoS_2_-loaded sponge evaporators. The MoS_2_ was securely attached to the sponge frame by means of an interlocking structure that relied on electrostatic attraction and the binding force of the binder. The final product’s low density allowed it to float on the surface of the water. The top layer, which contained the MoS_2_ coating, absorbed sunlight and converted it into heat, causing water to evaporate. On the other hand, internal capillary forces drew unheated water from the bottom layer to the upper layer.

**Figure 3:**
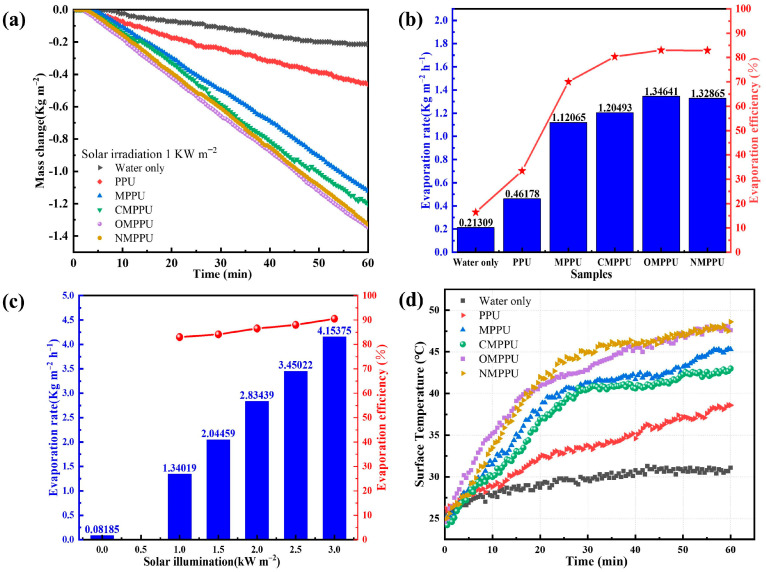
(**a**) Mass change over time of various evaporators under the illumination of 1.0 kW m^−2^. (**b**) Evaporation rates and the corresponding evaporation efficiency of multiple evaporators. (**c**) Evaporation rates and related efficiency under the illumination of 0, 1.0, 1.5, 2.0, 2.5, 3.0 kW m^−2^. (**d**) Temperature variations on the top surfaces of seawater, PPU, MPPU, CMPPU, OMPPU, and NMPPU evaporators as a function of time under the illumination of 1.0 kW m^−2^.

To examine the effect of photothermal evaporation under various experimental conditions, the evaporator was submerged in a glass containing 80 mL of a 3.5% NaCl solution. Figure 3a illustrates the change in saline mass over time that was observed. Observations revealed that the mass change was initially constant and enhanced linearly with time. Over time, the disparity in mass between the four MoS_2_-loaded evaporators grew substantially. Notably, the functionalized MoS_2_ evaporators demonstrated a greater change in water volume, with hydroxyl- and amino-functionalized MoS_2_ evaporators exhibiting the highest evaporation efficiency. This is because the modification effect enhances the hydrophilicity of MoS_2_ and the bonding effect with polyurethane sponge [50,51].

The evaporation rates and corresponding efficiencies of various evaporators were analyzed under a light intensity of 1 kW m^−2^, as depicted in Figure 3b. The evaporation rate of the PPU evaporator was 0.46 kg m^−2^ h^−1^, which increased significantly to 1.12 kg m^−2^ h^−1^ after loading with MoS_2_ (MPPU). Notably, the three functionalized MoS_2_ evaporators exhibited higher evaporation rates, with CMPPU at 1.20 kg m^−2^ h^−1^, OMPPU at 1.35 kg m^−2^ h^−1^, and NMPPU at 1.33 kg m^−2^ h^−1^. These results demonstrate the potential of functionalized MoS_2_ as an efficient evaporator for solar interfacial evaporation, with promising applications in water treatment and desalination. The evaporation efficiency of the solar evaporation process was calculated using Equations (2) and (3), and the results are presented in Figure 3b. The MoS_2_ evaporator exhibited an evaporation efficiency of 70.0%. In contrast, the CMPPU, OMPPU, and NMPPU evaporators demonstrated significantly higher evaporation efficiencies of 80.4%, 83.0%, and 82.8%, respectively. The functionalization process led to a substantial improvement in both the evaporation rate and efficiency of the evaporators. Further investigations on OMPPU were conducted owing to its superior evaporation rate and efficiency.

As presented in Figure 3c, the evaporation rates of the OMPPU evaporator under irradiation at 1.0, 1.5, 2.0, 2.5, and 3.0 kW m^−2^ are 1.34, 2.04, 2.83, 3.45, and 4.15 kg m^−2^ h^−1^, respectively. These values are 16.3, 24.8, 34.4, 41.9, and 50.3 times higher than the natural evaporation rate of seawater in dark conditions, which is 0.082 kg m^−2^ h^−1^. The MPPU evaporator exhibited an evaporation efficiency of 83.0% under low light conditions of 1.0 kW m^−2^, and over 90.5% under high solar levels (3 kW m^−2^). It is demonstrated that the OMPPU evaporator significantly enhances the evaporation rate, making it a promising solution for efficient solar thermal steam generation.

Figure 3d displays the temporal surface temperature profiles of evaporator-less, PPU, MPPU, CMPPU, OMPPU, and NMPPU evaporators exposed to 1.0 kW m^−2^ illumination. The top surfaces of the OMPPU and NMPPU evaporators maintained high temperatures (~50 °C), consistent with the previous evaporation rate and efficiency results.

**Figure 4:**
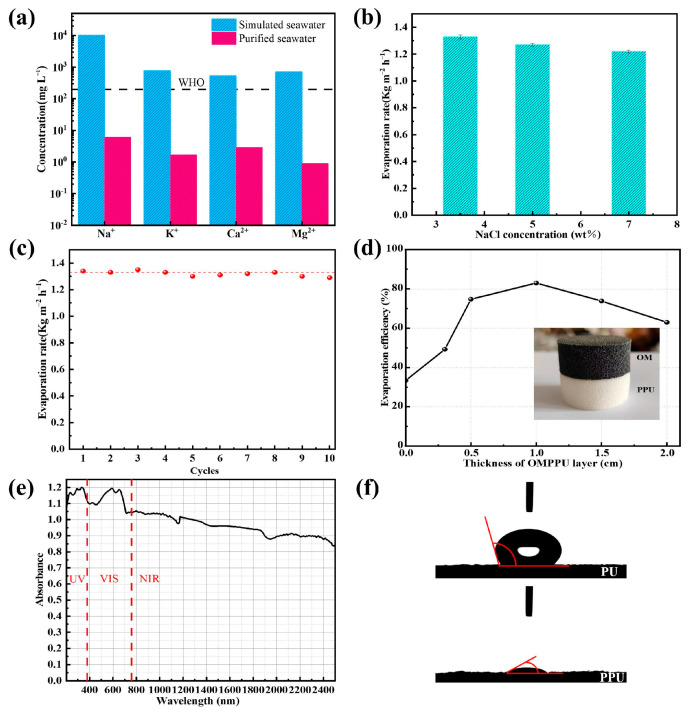
(**a**) The concentrations of different ions in simulated seawater and purified water (**b**) Evaporation rate of OMPPU under different brine concentrations. (**c**) Evaporation rate of OMPPU in 10 cycles. (**d**) Effect of the thickness of OM layer on the evaporation efficiency of OMPPU evaporators. (**e**) Solar absorption property of the OMPPU. (**f**) Contact angles of PPU before and after the function of PVA.

In Figure 4a, the results of the analysis conducted on the clean water produced by the evaporation process are illustrated. Initially, seawater had ion concentrations of Na^+^, K^+^, Ca^2+^, and Mg^2+^ at 10,030, 756, 530, and 697 mg L^−1^, respectively; after the evaporation process utilizing the OMPPU evaporator, the concentration of these ions significantly decreased to 6.1, 1.69, 2.88, and 0.87 mg L^−1^, respectively. These values were well below the World Health Organization (WHO) standards for drinking water, which set limits at 200 mg/L [52]. The significant reduction in ion concentration in the water sample demonstrates that the OMPPU evaporator is an efficient method for producing pure water, as it removes salts from seawater.

Figure 4b depicts the alterations in mass for OMPPU while treating saline water with concentrations of 3.5 wt%, 5.0 wt%, and 7.0 wt%. As the concentration of salt escalates to 5.0 wt% and 7.0 wt%, the corresponding mass change declines, but the variance is not significant. This implies that OMPPU has broad applicability in treating brine with diverse salinity levels. Upon conclusion of evaporation, there was no apparent accumulation of salt on the surface of OMPPU at 3.5 wt% and 5.0 wt% concentrations. As the concentration increased to 7.0 wt%, some salt accumulation was evident on the edge of the OMPPU but not on the central surface. This was induced by the concentration gradient, and the accumulation at the edge did not significantly deteriorate the evaporation performance (Figure S4). In addition, the evaporation rates of OMPPU in treating 5.0 wt% and 7.0 wt% NaCl solutions were 1.27 kg m^−2^ h^−1^ and 1.22 kg m^−2^ h^−1^, respectively, which were both slightly lower than the evaporation rate of OMPPU in 3.5 wt% NaCl solution, confirming the wide applicability of OMPPU.

To evaluate the robustness and performance stability of the OMPPU structure, cyclic experiments were conducted. As depicted in Figure 4c, solar evaporation was conducted for two hours during each cycle, after which the evaporation unit was placed in a dark environment until the following day. The evaporation rate of seawater in the presence of OMPPU was determined to be 1.29 kg m^−2^ h^−1^, which was comparable to the evaporation rate of OMPPU when initially treating a 3.5 wt% NaCl solution. Remarkably, the morphology of OMPPU did not exhibit any indications of collapse (Figure S5), indicating that it possesses exceptional mechanical properties, a crucial aspect for practical applications.

A series of experiments were conducted to determine the effect of hydroxyl-functionalized MoS_2_ coating (OM) thickness (ranging from 0 cm to 2.0 cm) on evaporation efficiency. The results are shown in Figure 4d. As the thickness of the coating increased from 0 cm to 1 cm, the evaporation efficiency increased from 33.43 percent to 83 percent. However, increasing the thickness further decreased the evaporator’s efficiency, indicating that the optimal thickness is crucial. Notably, a coating thickness of 0.2 cm allowed light transmission through OMMPU, while a thickness of 1 cm produced the greatest results. As coating thickness increased beyond 1 cm, evaporation efficiency decreased to 63%, possibly due to the denser MoS_2_ coating impeding the upward transport of water. These findings on the optimal thickness of hydroxyl-functionalized MoS_2_ coating for evaporation performance provide ideas for the design of evaporators.

The ability of photothermal materials to absorb sunlight is crucial in determining their potential performance in solar evaporation. As depicted in Figure 4e, a UV-Vis-NIR spectrophotometer measurement was conducted to evaluate the light absorption capacity of OMPPU in the wavelength range (200–2500 nm). OMPPU displays significant solar absorption (>85%) between 800 and 2500 nm. These results indicate that nanoscale MoS_2_ in OMPPU can harvest solar energy efficiently across the entire solar spectrum, making it a promising material for solar evaporation applications. Figure 4f illustrates a polyvinyl alcohol-functionalized polyurethane sponge (PPU) that considerably enhances the sponge’s hydrophilicity, thereby facilitating the upward transport of water during evaporation. PPU hydrophilic causes salt to redissolve in water during evaporation, averting salt deposition effectively.

## 3. Experimental Sections

### 3.1. Materials

Sodium sulfate (Na_2_SO_4_), N-Methyl pyrrolidone (C_5_H_9_NO), Isopropanol (C_3_H_8_O), Ethanol (C_2_H_5_O), Thioglycolic acid(C_2_H_4_O_2_S), 1-Thioglycerol (C_3_H_8_O_2_S), Cysteamine (C_2_H_7_NS), Hydroxyethyl cellulose ((C_2_H_6_O_2_)_n_), and Carboxymethyl cellulose were supplied by Aladdin Bio-Chem Technology Co., Ltd., Shanghai, China. Waterborne polyurethane was provided by Macklin Bio-Chem Technology Co., Ltd., Shanghai, China. Polyvinyl alcohol ([-CH_2_CHOH-]n) and Sodium dodecyl benzenesulfonate (C_18_H_29_NaO_3_S) were supplied by Sinopharm chemical reagent Co. Ltd., Shanghai, China. Polyurethane sponge was purchased from Yongjia spongy manufactures, Ganzhou, China. All chemicals were of analytical purity.

### 3.2. Exfoliation of Molybdenite

#### 3.2.1. Electrochemical Method of Electrolysis of Molybdenite Flakes

Natural molybdenite was used as the cathode, platinum electrode as the anode, and 0.5 mol/L Na_2_SO_4_ as the electrolyte. Expanded molybdenite flakes were obtained after 12 h of electrolysis at 0.025 A current.

The optimal electrolysis conditions were determined by comparing the changes in molybdenite thickness after electrolysis under the influence of different electrolysis times, electrolysis current, and molybdenite thickness. The degree of expansion of molybdenite is expressed by calculating the expansion coefficient through the following equation:

(1) $$E=\frac{e_{2}-e_{1}}{e_{1}},$$

where $e_{1}$ is the thickness of molybdenite before electrolysis, $e_{2}$ is the thickness of molybdenite after electrolysis, and E is the expansion coefficient of molybdenite.

#### 3.2.2. Liquid Exfoliation of Expanded Molybdenite Flakes

For liquid exfoliation, selecting the appropriate exfoliation solvent [53] facilitates exfoliation when the surface tension of the solvent matches the surface energy of the laminated bulk material [54]. Put the expanded molybdenite flakes into a mortar and add isopropanol at a ratio of 3 mL/g and mix the molybdenite flakes with the isopropanol thoroughly by grinding. Then the milled molybdenite was added to a mixture of isopropanol: deionized water 1:1 solution, and the suspension above was collected by ultrasonic at 500 W for 2 h. MoS_2_ nanosheets were obtained after centrifugation at 10,000 rpm for 20 min, and the MoS_2_ nanosheets were collected after continued ultrasonic centrifugation of the lower deposited molybdenite layer 2–4 times. The absorption coefficient (ε) at 670 nm was tested by the Beer–Lambert law to compare the effects of molybdenite solutions with different initial concentrations, ultrasonic time, and ultrasonic power on the yield of MoS_2_ nanosheets (A/l = ε C) [55].

#### 3.2.3. Functionalization of MoS_2_

Three organic compounds containing sulfhydryl groups reacted with MoS_2_, taken 0.1 g MoS_2_ and 10 mL thioglycolic acid(C_2_H_4_O_2_S) solution in a 20 mL sample bottle, with 1-Thioglycerol(C_3_H_8_O_2_S) solution dissolved (0.8 mL) in 100 mL water, with 0.2 g Cysteamine (C_2_H_7_NS) added to 5 mL water, the three mixed solutions were sonicated for 2 h and then stirred magnetically for 24 h and repeatedly washed. The excess organic solvent was removed and then freeze-dried to obtain three functionalized MoS_2_, named CM, OM, and NM, respectively.

#### 3.2.4. Functional MoS_2_ Coating Preparation

Dissolve 1 g of MoS_2_ and three kinds of functionalized MoS_2_ in 100 mL of isopropanol: water 1:1 solution, then add 0.02 g of sodium dodecylbenzene sulfonate, and make it fully dispersed by supernatural for 30 min. Take 30 mL of the mixed solution and add 10 mL of water-based polyurethane emulsion, 30 mL of 2 g/100 mL of carboxymethyl cellulose as OM and NM thickener, 30 mL of 2 g/100 mL of hydroxyethyl cellulose as CM thickener, mix, and stir for 18 h to make functionalized MoS_2_ coating.

#### 3.2.5. Evaporator Preparation

The polyurethane sponge (PU) with a diameter of 4 cm and a thickness of 2 cm was selected as the substrate. The polyvinyl alcohol functionalized polyurethane sponge (PPU) was obtained by impregnating it in a 20 g/L polyvinyl alcohol solution, repeatedly pressing the sponge by hand to fully absorb the polyvinyl alcohol solution, and then drying it. The coating was applied to the PPU, unfunctionalized MoS_2_ was loaded on PPU by ultrasonic named MPPU, and the other three coating-coated evaporators were named CMPPU, OMPPU, and NMPPU. In addition, five different functionalized MoS_2_ loading thicknesses of 0.2 cm, 0.5 cm, 1.0 cm, 1.5 cm, and 2.0 cm were obtained by varying the amount of coating, and the effect of the different thicknesses on the evaporation efficiency was tested.

### 3.3. Characterization Methods

The X-ray Diffractometer (XRD) was analyzed by SmartLab SE, Rigaku, Japan. The morphology (SEM) of samples was obtained from Gemini 300, ZEISS, German. The Fourier-transformed infrared (FT-IR) spectra were recorded on Scientific Nicolet iS20, Thermo, USA in a transmittance mode in a wavenumber range of 4000 to 400 cm^−1^. The atomic force microscopy (AFM) measurement was performed on a Dimension ICON, Bruker, UK. in a tapping mode. The UV-visible NIR diffuse reflectance was tested by UV-3600, Shimadzu, Japan. The contact angles were measured with the pendant-drop method on OCA 20, Dataphysics, German. The concentration of ions in stimulated seawater and purified water was analyzed by NexION 1000 G/NexION 1000 G Inductively coupled plasma mass spectrometer (ICP-OES), PerkinElmer, USA.

### 3.4. Experiments of Solar Steam Generation

The solar simulator (PLS-SXE300/300UV, Perfectlight, Beijing, China), which was regulated to have a modest solar density of 0–3 kW m^−2^, provided broadband lighting. The Perfectsolar PL-MW2000(Perfectlight, Beijing, China) photoradiometer was used to gauge solar intensity. To monitor the changes in water mass in real time, an electronic balance (Shanghai Yueping, YP6002, accuracy: 0.01 g) was set up with a beaker holding the floating evaporator and saline water. A probe from an electronic temperature logger (GSP-6, Jingchuang, Jiangsu, China) was placed into the top layer of the evaporators to record the temperature vibration. The ambient temperature was maintained at 23 ± 2 °C by the air conditioner throughout all of the sun desalination trials. The evaporation rate (*ν*) was calculated using the equation:

(2) $$\dot{m}=\frac{\Delta m}{\Delta t•A},$$

where ṁ is the stable evaporation rates (ṁ = ṁ_Light_ − ṁDark), ṁ_Light_ and ṁDark are the evaporation rate under light and dark conditions, respectively [56], A is the surface of the evaporator, and Δt is the test time.

(3) $$\eta =\dot{m}•\frac{\Delta_{vap}H_{m}+C\Delta T}{C_{opt}q_{i}},$$

where ΔvapHm represents the phase-change enthalpy of liquid-steam and CΔT is sensible heat. C is a constant value (4.18 J g^−1^ k^−1^), ΔT is the different of steam evaporation (*T*_1_), and room temperature (~25 °C), $q_{i}$ means the normalized solar intensity, 1.0 kW m^−2^; $C_{opt}$ is the multiple of 1.0 kW m^−2^ [57].

## 4. Conclusions

A novel and facile method of MoS_2_ double-layer evaporators for solar interfacial desalination are demonstrated in this work. The functional groups were introduced on the surface of MoS_2_ through the combination of sulfhydryl groups and S vacancies. Functionalization enables MoS_2_ to be firmly bonded to polyurethane sponge for the purpose of enhancing the evaporation effect. Among them, the hydroxyl functionalized MoS_2_ evaporator has the best evaporation effect, with an evaporation rate of 1.35 kg m^−2^ h^−1^ and an evaporation efficiency of 83% at one sun. The evaporators possessed a porous structure, excellent chemical and mechanical properties, and great solar light absorption capacity. The functionalized MoS_2_-based coating provides a scalable method for solar interfacial desalination.

## Data Availability

Not applicable.

## Supplementary Materials

The following supporting information can be downloaded at: https://www.mdpi.com/article/10.3390/ma16083105/s1, Figure S1: Effect of current (a) and time (b) on expansion rate; Figure S2: SEM images of MoS_2_ supported on PU sponge by ultrasonic; Figure S3: Optical image of the flexible evaporators (Layer thickness: MPPU: 0.5 cm, PPU: 0.5 cm); Figure S4: The digital images of salt accumulation on the surface of OMPPU after one night of dark treatment; Figure S5: Optical images of the top surface of OPPU at the (a) beginning and (b) after 20 h; Table S1: Three condition orthogonal experimental response surface; Table S2: Optimal condition selection.
