# Supplementary material for: A Novel Functionalized MoS2-Based Coating for Efficient Solar Desalination
Source: Materials (Basel). 2023 Apr 14;16(8):3105. doi: 10.3390/ma16083105 (PMC10141543; doi:10.3390/ma16083105)
Supplement: Supplementary file 1 [file materials-16-03105-s001.zip › materials-2335474-supplementary.pdf]

A novel functionalized MoS_2-based coating for efficient solar desalination

Qinghong, Yu[1,2], Qingmiao Wang[1,2*], Tao Feng[1,2*], Li Wang[1,2], Zhixuan, Fan[1,2]

[1] College of Resources and Environmental Engineering, Wuhan University of Science and Technology, Wuhan, Hubei Province, 430081, China.

[2] Center of Green Control and Remediation Technologies for Environmental Pollution, Wuhan University of Science and Technology, Wuhan, Hubei Province, 430081, China.

Corresponding authors: (Q. Wang) qingmiaowang@wust.edu.cn； (T. Feng) fengtaowhu@163.com

		Factor 1	Factor 2	Factor 3	Response 1
Std	Run	A: Time(h)	B: Current（A）	C: Thickness (mm)	Expansion Coefficient
9	1	12	0.02	0.5	4.38
6	2	14	0.025	0.5	5.02
12	3	12	0.03	2	1.61
13	4	12	0.025	1.25	3.64
14	5	12	0.025	1.25	3.968
7	6	10	0.025	2	1.535
10	7	12	0.03	0.5	5.02
4	8	14	0.03	1.25	3.56
5	9	10	0.025	0.5	4.22
8	10	14	0.025	2	1.915
2	11	14	0.02	1.25	3.296
11	12	12	0.02	2	1.445
16	13	12	0.025	1.25	3.824
3	14	10	0.03	1.25	3.224
15	15	12	0.025	1.25	3.728
17	16	12	0.025	1.25	3.712
1	17	10	0.02	1.25	2.984

Text 1 Three condition orthogonal experimental response surface

**Time(h)**	**Current(A)**	**Thickness(mm)**	**Expansion Coefficient**	**Desirability**	
**12.884**	**0.025**	**0.518**	**5.021**	**1.000**	**Selected**

Text 2 Optimal condition selection

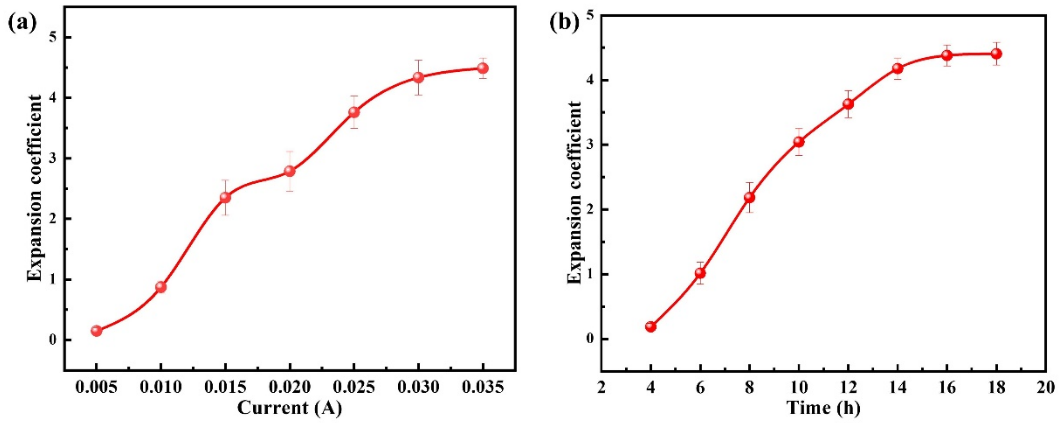

Figure S1. Effect of current（a）and time（b）on expansion rate.

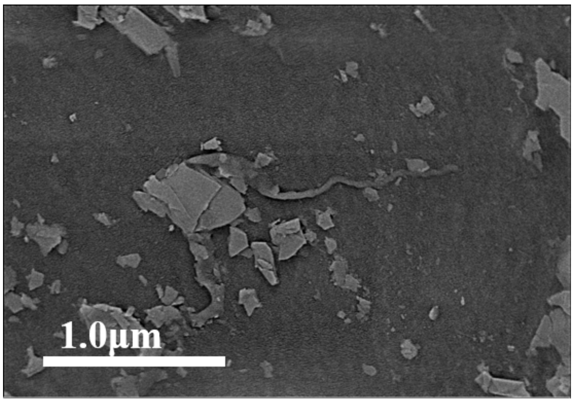

Figure S2. SEM images of MoS_2 supported on PU sponge by ultrasonic

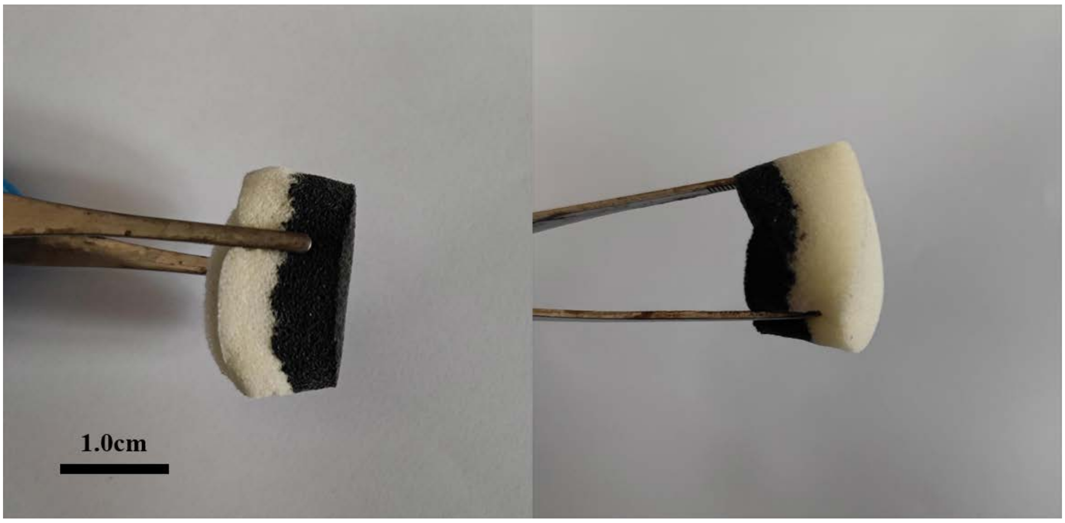

Figure S3. Optical image of the flexible evaporators (Layer thickness: MPPU: 0.5 cm, PPU: 0.5 cm).

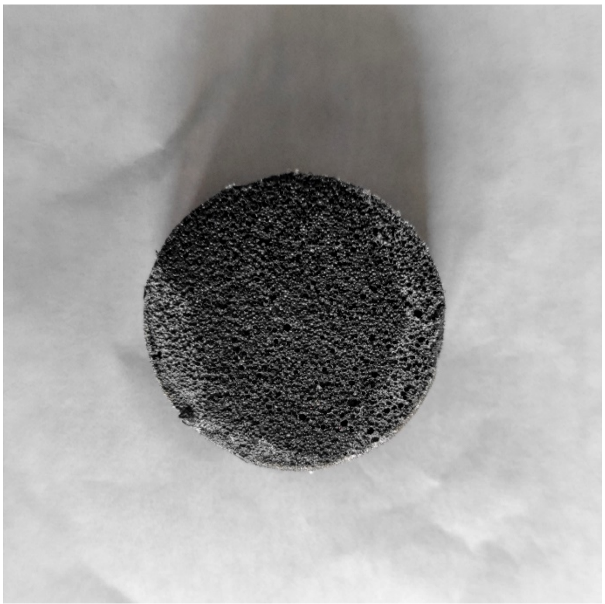

Figure S4. The digital images of salt accumulation on the surface of OMPPU after one night of dark treatment.

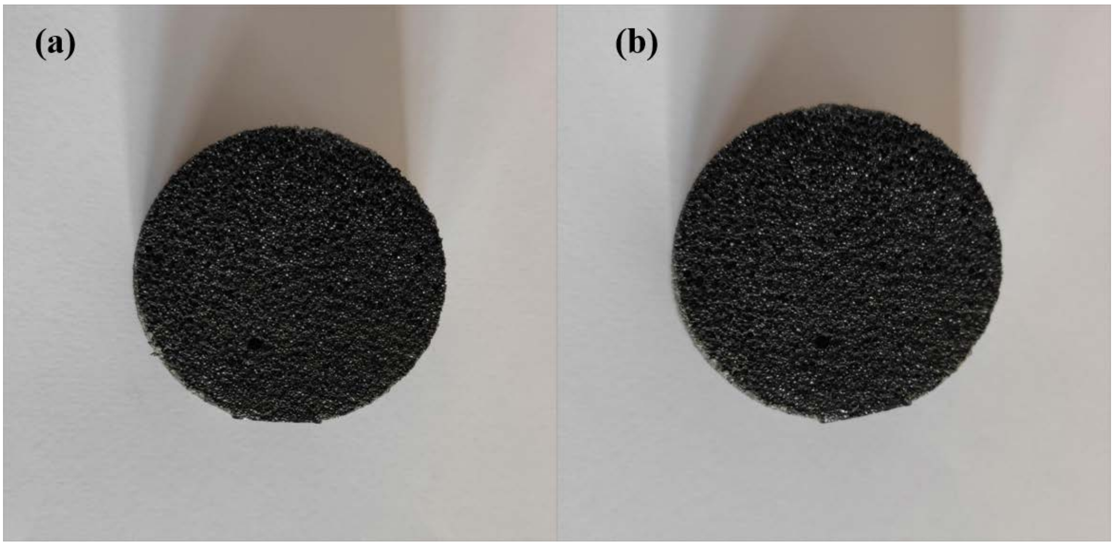

Figure S5. Optical images of the top surface of OPPU at the（a）beginning and (b) after 20 hours
